# Control of HCV Replication With iMIRs, a Novel Anti-RNAi Agent

**DOI:** 10.1038/mtna.2014.71

**Published:** 2015-01-20

**Authors:** Saori Itami, Yutaka Eguchi, Takayuki Mizutani, Eriko Aoki, Tadaaki Ohgi, Masahiko Kuroda, Takahiro Ochiya, Nobuyuki Kato, Hiroshi I Suzuki, Norifumi Kawada, Yoshiki Murakami

**Affiliations:** 1 Department of Hepatology, Graduate School of Medicine, Osaka City University, Osaka, Japan; 2 Department of Medical Genetics, Osaka University Graduate School of Medicine, Suita, Japan; 3 BONAC Corporation, Kurume, Japan; 4 Department of Molecular Pathology, Tokyo Medical University, Tokyo, Japan; 5 National Cancer Center Research Institute, Tokyo, Japan; 6 Department of Tumor Virology, Okayama University, Okayama, Japan; 7 Sharp Laboratory Koch Institute for Integrative Cancer Research, Massachusetts Institute of Technology, Cambridge, Massachusetts, USA

**Keywords:** anti-sense oligonucleotide, hepatitis C virus, interferon, locked nucleic acid, miRNA, RNA interference

## Abstract

MicroRNAs (miRNAs) serve important roles in regulating various physiological activities through RNA interference (RNAi). miR-122 is an important mediator of RNAi that is known to control hepatitis C virus (HCV) replication and is being investigated in clinical trials as a target for anti-HCV therapy. In this study, we developed novel oligonucleotides containing non-nucleotide residues, termed iMIRs, and tested their abilities to inhibit miR-122 function. We compared the inhibitory effects of iMIRs and locked nucleic acids (LNAs) on HCV replication in OR6 cells, which contained full-length HCV (genotype 1b) and a luciferase reporter gene. We found that RNA-type iMIRs with bulge-type, imperfect complementarity with respect to miR-122 were 10-fold more effective than LNAs in inhibiting HCV replication and functioned in a dose-dependent manner. Moreover, iMIR treatment of OR6 cells reduced HCV replication without inducing interferon responses or cellular toxicity. Based on these results, we suggest that iMIRs can inhibit HCV replication more effectively than LNAs and are therefore promising as novel antiviral agents.

## Introduction

RNA interference (RNAi) is a cellular mechanism that mediates gene silencing in a sequence-specific manner.^1,2,3^ MicroRNAs (miRNAs) are endogenous small noncoding RNAs that control gene expression by degrading or suppressing the translation of target mRNAs.^4^ Approximately 70% of the total miRNA expressed in liver cells is miR-122.^5^ miR-122 accelerates hepatitis C virus (HCV) replication.^6^ Targeting multiple viral and cellular elements involved in RNAi may increase the potency of antiviral gene therapies,^7,8^ or find clinical applications in eradicating persistent infections.

HCV infection is the leading cause of chronic hepatitis (CH), liver cirrhosis, and hepatocellular carcinoma (HCC). Several direct-acting antiviral agents (DAAs) have been developed to combat HCV. Among these DAAs, simeprevir and asunaprevir inhibit the HCV NS3 protease, daclatasvir and ledipasvir inhibit NS5A, and sofosbuvir inhibits NS5B.^9^ The addition of simeprevir to standard chemotherapy with pegylated interferon (Peg-IFN) and ribavirin markedly improved curative effects, as compared to results obtained with Peg-IFN and ribavirin therapy alone.^10^ In general, the side effects of anti-HCV therapy are mostly attributable to IFN, which may explain why several side effects were reduced in patients treated with regimens lacking IFN. However, two major drawbacks were uncovered for IFN-free therapy. The first is that the curative effects vary according to HCV genotype. The second is the emergence of drug-resistant variants. Thus, despite existing and investigative therapies, there is still an urgent need to develop alternative CHC treatments that result in fewer side effects.

Recently, nucleic acid medicine has emerged as a potential treatment strategy for CHC with enhanced efficacy and fewer adverse effects. A locked nucleic acid (LNA)–modified DNA phosphorothioate antisense oligonucleotide (ASO) that sequesters mature miR-122 in a highly stable heteroduplex inhibited its function with no dose-limiting adverse events and no escape mutants generated in the miR-122 binding sites of the HCV genome. The use of this nucleic acid against CHC genotype 1 infection resulted in prolonged, dose-dependent reductions in HCV levels without evidence of viral resistance.^11^ Besides miR-122, several alternative nucleic acid therapies will be tested in clinical trials.^12,13,14^

Several obstacles need to be overcome before nucleic acid medicine can be applied clinically. One such obstacle is that siRNAs or miRNAs are promptly degraded by nucleases in the serum and/or extracellular fluids when they are administered systemically.^15,16^ Chemical modifications at specific positions may help to improve stability, but these modifications may attenuate the suppressive activity of siRNAs.^17^ The cost of producing nucleic acid-based therapeutics on a large-scale is another hindrance to the clinical application of chemically modified or unmodified siRNA agents.^18^ With these disadvantages in mind, it is clear that additional research is crucial to fully develop and utilize nucleic acid therapies.

In this study, we successfully controlled HCV replication *in vitro* using novel anti-RNAi agents, termed iMIRs. We demonstrated that specific iMIRs exert RNA silencing-based antiviral responses during HCV replication without cellular toxicity. We suggest that the approach presented here may be applied to miRNA-mediated gene control.

## Results

### iMIR construction and HCV reporter gene assay

**Figure 1:**
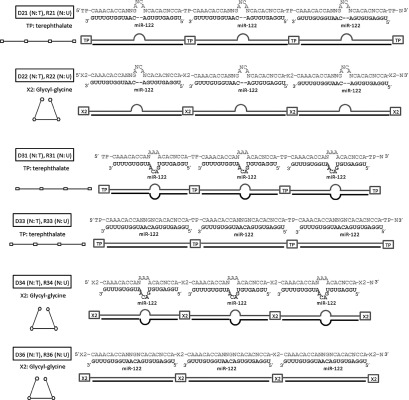
**Structure of iMIRs**. The complementarity between iMIRs and the target miR-122 sequence is shown schematically. The primary sequences of iMIRs and miR-122 are shown in gray and black text, respectively. D and R symbolize DNA-based iMIRs (N, thymidine) and RNA-based iMIRs (N, Uracil), respectively. In each case, the three miRNA-binding sequences were connected by non-nucleotide compounds, either terephthalate (TP) or glycyl-glycine (X2), and both ends of the molecules were protected with the same compounds (either TP or X2). Their schematic constructions of iMIR are shown in the left panel. The squares represent terephthalate groups, the hexagons represent glycyl-glycine groups, and the lines show the miRNA-binding nucleotide sequence.

We developed novel agents, termed iMIRs, to inhibit miRNAs. The iMIR nucleotides used in this study were composed of 3 identical miR-122 binding sites joined in tandem. iMIRs were designed as either DNA or RNA molecules that perfectly matched miR-122 or that formed a bubble-type or bulge-type mismatch. To validate the structural formation, fragments were linked by two non-nucleotide residues (terephthalate or glycyl-glycine), and same residues were attached at the ends of fragment. These iMIRs were numbered and abbreviations are shown in **Figure 1**. For example, D21 means DNA-type sequence, bulge-type complementary to miR-122, linked by terephthalate. The effect of iMIRs on HCV replication was assessed in HCV replicon cells (OR6), which contain the full-length HCV genome (genotype 1b) and a Renilla luciferase reporter gene.^19^

### iMIR can control miR-122 function

**Figure 2:**
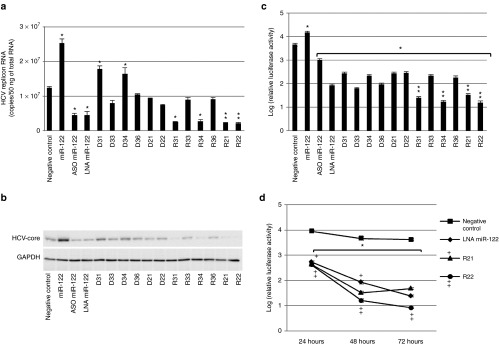
**Inhibition of hepatitis C virus (HCV) replication by several anti-RNAi agents**. (**a**) Effects of miR-122–specific oligonucleotides on HCV replicon RNA levels in OR6 cells. HCV replicon RNA copy numbers per 50 ng of total RNA were measured by real-time quantitative polymerase chain reaction (qPCR) at 48 hours post-transfection with a double-stranded (ds) mature miR-122, an miR-122 antisense oligonucleotide (ASO), an miR-122 locked nucleic acid (LNA), candidate miR-122 iMIRs, and a negative control for miR-122 (40 nmol/l). The data shown are mean ± SD values of three independent experiments. Single asterisks denote significant differences from the negative control (*P* < 0.05). Double asterisks denote significant differences from the miR-122 LNA (*P* < 0.05). (**b**) Immunoblot analysis of HCV core proteins and GAPDH (internal control) in whole OR6 cell lysates transfected with ds-miR-122, miR-122 ASO, miR-122 LNA, iMIRs, or a negative control siRNA (40 nmol/l). Whole cell lysates (50 μg) harvested at 48 hours after transfection were analyzed. (**c**) Relative luciferase activities in OR6 cell lysates cotransfected for 48 hours with a pGL3 control vector (1 μg/ml) encoding firefly luciferase and either ds-miR-122, ASO-miR-122, LNA-miR-122, iMIRs, or negative control siRNA (40 nmol/l). Firefly luciferase activities were normalized to *Renilla* luciferase activity. Single asterisks denote significant differences from the negative control at *P* < 0.05. Double asterisks denote significant differences from the miR-122 LNA at *P* < 0.05. (**d**) Inhibition of HCV replication by iMIRs in time course assays. Time-course assays demonstrating inhibition of HCV replication by iMIRs (40 nmol/l). Relative luciferase activities in lysates from OR6 cells cotransfected with the pGL3-control vector and either LNA-miR-122, iMIRs, or a negative control siRNA for 24, 48, or 72 hours. Renilla luciferase activities were normalized to firefly luciferase activities. The asterisk denotes a significant difference from the negative control (*P* < 0.05). Where indicated, the “+” and “++” signs denote significant differences in luciferase activities (*P* < 0.05) between miR-122 LNA transfectants and R21 or R22 transfectants, respectively.

The effects of miR-122, an LNA-miR-122, and an ASO-miR-122 on HCV replication were measured by real-time quantitative polymerase chain reaction (qPCR) (**Figure 2a**), by immunoblots against the HCV core protein (**Figure 2b**), and by dual-luciferase reporter gene assays (**Figure 2c**). We observed that transfecting mature miR-122 into OR6 cells significantly accelerated HCV replication. Conversely, inhibiting miR-122 expression by transfection with either an LNA-miR-122 or an ASO-miR-122 significantly inhibited HCV replication (**Figure 2a**–**c**).

To evaluate the antiviral activity of iMIRs, each of the variably designed iMIR constructs (**Figure 1** and **Table 1**) was transfected to OR6 cells, and their effects on HCV replication were monitored. We observed that RNA-type iMIRs had a tendency to inhibit HCV replication more effectively than DNA-type iMIRs (**Figure 2a**–**c**).

The R21 and R22 iMIRs significantly inhibited HCV replication, and their inhibitory functions were more effective than LNA-miR-122. Although R21 and its DNA-based analog, D21, (and also R22 and D22) share the same complementarity and similar hybridization structures with miR-122, D21 and D22 did not significantly suppress HCV replication. R31 and R34 inhibited HCV replication more effectively than LNA-miR-122, although D31 and D34 did not. R33, D33, R36, and D36 did not suppress HCV replication, indicating that iMIRs with perfectly matched sequences against the target miR-122 were less effective, regardless of whether they were composed of RNA or DNA bases (**Figure 2a**). In luciferase assays, all iMIRs significantly repressed the expression of luciferase, which represented the efficiency of HCV replication, and the suppressive effects of R31, R34, R21, and R22 was significantly greater than that of LNA-miR-122 (**Figure 2c**). In addition, we noted the iMIR side chains did not influence the inhibition of HCV replication. The remarkable ability of R21 or R22 to inhibit HCV replication was verified by western blots (**Figure 2b**). To confirm the stability of iMIR activity, HCV replication in OR6 cells was examined in luciferase time-course assays (**Figure 2d**). At 24, 48, and 72 hours post-transfection, HCV replication was inhibited by LNA-miR-122 and iMIRs (R21 and R22). R21 and R22 inhibited HCV replication significantly more than LNA-miR-122 at 24 and 48 hours. In addition, R22 suppressed HCV replication significantly more than LNA-miR-122 at all the time points examined. The effects of iMIRs on HCV replication continued from 24 to 72 hours, indicating that the iMIRs functioned for a long period in cells.

**Figure 3:**
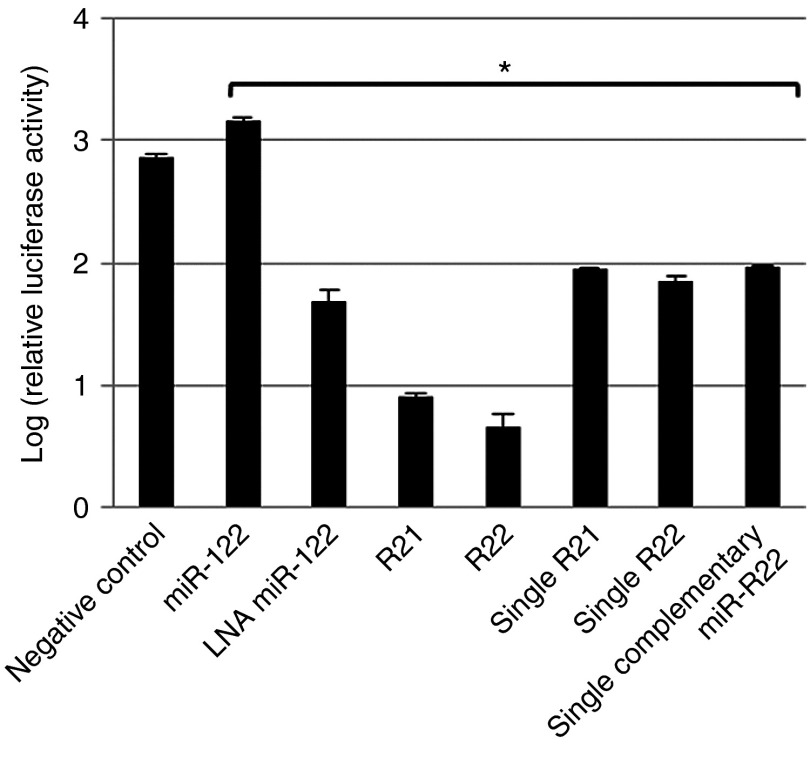
**Comparison of the inhibition of HCV replication by a miR-122 LNA or single- and double-stranded iMIRs**. Comparison of HCV replicon RNA levels in OR6 cells transfected with ds-miR-122, 40 nmol/l of LNA-miR-122, R21, R22, single R21, single R22, or a single miR-122 without amino acid modification. Relative luciferase activities are shown. The data shown are mean ± SD values from three independent experiments. The asterisk denotes significant differences from the negative control (*P* < 0.05).

To verify the structural advantages of iMIRs, we prepared oligonucleotide-carrying monomer units of bulge-type miRNA-binding sequences (MBSs) derived from R21 and R22, whose ends were protected with terephthalate (single R21) or glycyl-glycine (single R22), and those without end-protection (single miR-122). HCV RNA replication was not suppressed by single R21 or single R22 as it was by R21 or R22 (**Figure 3**), suggesting that the tandem configuration of triplicated MBSs may be responsible for higher iMIR activity.

**Figure 4:**
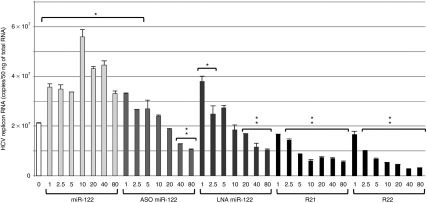
**Dose-dependent inhibition of hepatitis C virus (HCV) replicon RNA production by anti-RNAi agents**. The amount of HCV replicon RNAs produced in OR6 cells transfected with various quantities (1, 2.5, 5, 10, 20, 40, and 80 nmol/l) of ds-miR-122, ASO-miR-122, LNA-miR-122, R21, and R22 was monitored by real-time quantitative polymerase chain reaction (qPCR). The data shown are mean ± SD values of three independent experiments. Single and double asterisks indicate values that were significantly higher or lower (*P* < 0.05) than the negative control, respectively.

As shown in **Figure 4**, R21 and R22 controlled HCV replication in a dose-dependent manner. Furthermore, a one-tenth concentration of iMIR was more effective at inhibiting HCV replication than a full concentration of LNA-miR-122. Although ASO-miR-122 and LNA-miR-122 also suppressed HCV replication in a dose-dependent manner, the effect was weak compared with iMIRs (**Figure 4**).

### The antiviral effects of iMIRs are independent of the IFN pathway

**Figure 5:**
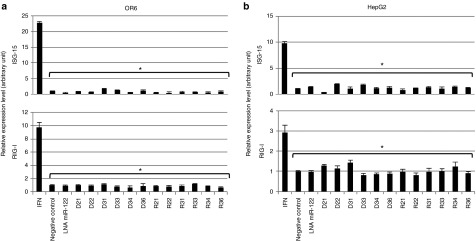
**Negligible induction of interferon (IFN)-related genes**. iMIRs can inhibit hepatitis C virus (HCV) replication independently of the interferon pathway. The expression levels of ISG15 (upper panel) and RIG-I (lower panel) in OR6 cells (**a**) and HepG2 cells (**b**) treated with 40 nmol/l of iMIRs for 48 hours were monitored by real-time quantitative polymerase chain reaction. Asterisks denote significant differences (*P* < 0.05) relative to ISG15 or RIG-1 induction observed in cells treated with 10^3^ U/ml IFN for 48 hours.

To clarify if the IFN pathway was involved in the inhibitory effects of iMIRs on HCV replication, we examined if the IFN pathway was induced by iMIR treatment. The expression levels of IFN-related genes (ISG15 and RIG-I) in OR6 and HepG2 transfected for 48 hours with iMIR were compared to positive-control cells treated with IFN. We found that treatment with IFN effectively induced ISG15 and RIG-I expression, while their expression levels in iMIR-treated cells were not significantly different from those found in negative-control and LNA-miR-122-treated cells (**Figure 5**).

### iMIRs did not induce toxicity in OR6 cells

**Figure 6:**
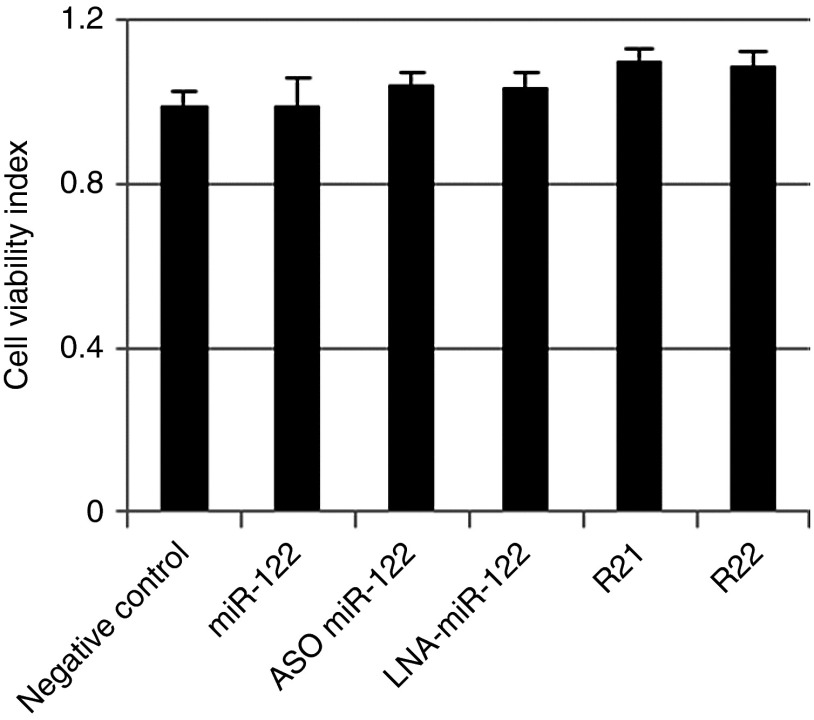
**Measurement of cellular toxicities in iMIR transfectants**. Cell proliferation indexes in OR6 cells transfected for 72 hours with the indicated iMIRs (40 nmol/l final concentration). The data shown are mean ± SD values of three independent experiments.

To determine whether the inhibitory effects of iMIRs resulted from cell toxicity, we examined the cell viability of OR6 cells after transfection with a negative control, mature miR-122, ASO-miR-122, LNA-miR-122, R21, and R22. XTT cell proliferation and viability assays were used to test for cytotoxicity. At 72 hours post-transfection, the cell proliferation index indicated by XTT was not significantly affected by any treatments (**Figure 6**), showing that transfecting OR6 cells with R21 or R22 did not generate cytotoxicity.

## Discussion

This study was designed to explore the effectiveness of iMIRs as novel anti-RNAi agents. Previously, several chemical modifications to improve anti-miRNA efficiency have been described.^20,21,22,23^ In the iMIR molecules tested in this study, all miRNA-identification sites were composed of natural nucleotides. The reasons for this design strategy were threefold. First, we sought to focus initially on presenting the fundamental characteristics of iMIRs without modifications using sequences targeting miRNAs. Second, when targeting chronic diseases, the accumulation of non-natural compounds in cells may cause toxicity, and by using natural nucleotides, it is possible that they may be reused in salvage pathways. Finally, the sites that did not affect miRNA inhibition or that resulted in a structural advantage (spacer site) were minimally modified compared to small natural compounds. Therefore, we first examined iMIR molecules without chemical modifications at miRNA binding sites. To reduce the interference between miR-122 binding sites, terephthalate bases (induced linear structure) and natural dipeptides (induced specific folding structure) were used, especially in the spacer sites. In addition, these residues were attached to both edges of iMIRs. Placing these residues at the ends of iMIRs increased the stability of the iMIRs. As shown in **Supplementary Figure S1**, both iMIR and single iMIR carrying these residues at both ends were not degraded by exonuclease T, while a single oligonucleotide complementary to miR-122 lacking these residues was degraded by the same treatment. To achieve the goal of using natural components, we developed novel amidites from amino acids and low molecular weight compounds using simple chemistry, which were optimized for solid-phase nucleotide synthesis. We showed that iMIRs made from natural materials could inhibit HCV replication more effectively than LNAs. The effects of iMIRs lasted for at least for 3 days, suggesting that iMIRs can function stably in cells for long durations. These characteristics might be improved by introducing nontoxic modifications to nucleotides and non-nucleotide residues, and further investigations should be performed to optimize iMIR function.

In this study, we also tested iMIRs and control molecules synthesized with phosphodiester-containing oligonucleotides. Phosphorothioate analogs have been used previously for the development of antisense nucleic acids with increased resistance to enzymatic activity. However, potential risks may manifest when using phosphorothioates, such as cell toxicity due to sulfur molecules and the nonspecific adsorption of proteins, factors that have not been fully investigated. Therefore, we used phosphodiester versions of iMIRs initially. The inhibitory effects of iMIRs on HCV replication were stronger than that of LNAs, and cell toxicity was not observed.

To evaluate and compare the effect of iMIRs with LNAs, we used miR-122 and HCV replicon cells. R21 and R22, both of which are made from RNA with bulge-type complementarity, were the most effective in suppressing miR-122 function. The effectiveness of iMIRs was evaluated in terms of the following three structural characteristics. First, three series of oligonucleotides were prepared with same MBS sequences bound by terephthalate or glycyl-glycine. R21 and R22 prepared with terephthalate and glycyl-glycine as linkers, respectively, showed similar inhibitory effects on HCV replication, suggesting that the length, but not the angle, might be important for linker molecules. Although the iMIRs presented in this study showed marked antiviral activities, further studies might be necessary for optimizing the distance between the MBS sequences. Second, three patterns of complementarity were investigated, namely perfect and imperfect matches (bubble and bulge sequences). We found that iMIRs with mismatches in the middle region of the MBS had higher activity than iMIRs that were perfectly matched to the target miRNA. Because iMIRs that had no complementary to miRNA did not effectively inhibit miRNA (data not shown), we surmise that the mismatched regions in the middle of MBS may serve a significant purpose. One possibility is that iMIRs with perfect complementarity could serve as a substrate for RNA-induced silencing complexes containing the target miRNA, thereby resulting in iMIR degradation. It was shown that microRNA sponges, forming bubble-type complementarity with miRNA sequences, or TuD RNA with bulge-type complementarity, could inhibit miRNA functions more than perfectly matched RNAs.^24,25,26^ In agreement with these reports, the mismatch in the middle of the MBS may prevent iMIR degradation. We prepared nucleotide composition as DNA or RNA. We expected that RNA-based iMIRs would show higher inhibitory effects on miRNA function than would DNA-based iMIRs because RNA binds more strongly to complementary RNA than to complementary DNA. In agreement, RNA-type iMIRs against miR-122 were much more effective than DNA-type iMIRs. In our preliminary experiments, we found that one of the DNA-type iMIRs functioned more effectively than the RNA-type iMIRs (data not shown). Thus, it is possible that the target miRNA and cell type may influence whether RNA-type or DNA-type iMIRs have higher efficiency. Using miR-122 and OR6 cells, RNA-type iMIRs were more effective than DNA-type in blocking miR-122 function, suggesting that they may be more promising as candidates for therapeutic drug development against HCV.

The antiviral effects of iMIRs presented in this study are likely due to a direct inhibitory action against miR-122, considering that the effects depended on the degree of sequence complementarity. We also showed that the IFN pathway was not involved in mediating the inhibitory effects of iMIRs on HCV replication and that iMIRs did not induce cytotoxicity, suggesting that the function of iMIRs were not derived from nonspecific toxic side effects on cells. In addition, iMIRs did not stimulate Toll-like receptors (TLR3, TLR7, and TLR9), as HEK293 cells overexpressing these TLRs did not respond to iMIRs (data not shown). Thus, iMIRs are predicted to be safe and specific agents.

Because iMIRs have three MBSs, it is possible that one molecule of the iMIR binds to two or more miRNAs. We found that even at a one-third concentration, iMIRs showed a higher capacity to inhibit miRNA than did other anti-miRNA agents with a single MBS, such as ASOs and LNAs. However, the inhibitory effects on miRNA function cannot be determined solely by the number of MBSs. In addition, the configuration of tandem MBSs in iMIR molecules seems to provide structural advantages for its activity. Since the activity of iMIR is maintained through at least 72 hours post-transfection, the binding ability of miRNAs and iMIRs may be more stable than other nucleic acids. Moreover, because the inhibitory effects of iMIRs on HCV replication were 10-fold greater than those of LNAs, iMIRs should show activity at increased durations post-transfection. Although iMIRs are relatively long oligonucleotides, than can be synthesized with simple and inexpensive amidites, and when inhibiting HCV replication,lowerdoses of iMIRs were required compared to LNAs, indicating that iMIRs may be affordable and advantageous over other methods of inhibiting miRNAs.

The expression of several miR-122-regulated host genes has been implicated in the development of hepatocellular carcinoma (HCC). Although a direct causal relationship between the sustained loss of miR-122 function and HCC remains to be determined, downregulation of miR-122 has been described in HCC, with lower miR-122 levels correlating with a poor prognosis.^27,28,29,30^ miR-122 maintains hepatic function by down-regulating genes involved in cholesterol synthesis, such as HMG-CoA reductase.^31^ Therefore, controlling the function of miR-122 for extended periods of time may induce numerous side effects or liver dysfunction. For example, miR-122 has been implicated in liver transformation, inflammation, and lipid metabolism.^32,33^ Additionally, the circadian metabolic regulators of the PPAR family are regulated by miR-122-mediated metabolic control.^34^ Thus, artificial manipulation of miR-122 expression may result in adverse effects. Janssen *et al.*^11^ showed that LNA medication results in few side effects because the degree of modulation of most miR-122-regulated target mRNAs is relatively small. In contrast, the effects of miR-122 sequestration appear to result in significant changes in HCV RNA levels.

A sustained viral response has been achieved in several patients treated with an IFN-free regimen that is combined with DAA.^35,36,37^ Although a dual DAA regimen without IFN resulted in better treatment outcome than combined IFN and ribavirin therapy, DAAs perform differently against each HCV genotype and can induce drug resistance that is associated with viral mutations.^38^ In contrast, the binding sites of miR-122 in the HCV genome are highly conserved, allowing for the use of LNAs or iMIRs targeting miR-122 in all HCV genotypes.^39^ Lanford *et al.*^40^ reported evidence of escape mutations in HCV RNA in primates and humans treated with LNA-miR-122, indicating a high genetic barrier to resistance. Based on this evidence, development of resistance to treatment can be expected when using miRNA-related regimens. Moreover, because antisense therapy is widely applicable, the strategy we have described here may also be relevant for diseases other than chronic HCV infection. Within the field of hepatology, the inhibition of miR-122 has been associated with an improvement in steatosis in a mouse model of diet-induced obesity, suggesting a role for miR-122 antagonism in the treatment of nonalcoholic fatty liver disease.^41^ Within other fields, therapeutic silencing of disease-associated miRNAs in preclinical studies of cancer and cardiovascular and autoimmune disorders has delivered results that warrant clinical investigation.^42,43,44^ Taken together, it is expected that iMIR treatment can reduce the frequency of side effects better than not only LNA-miR-122, but also IFN and DAAs as well.

This report described the *in vitro* properties of iMIR, a novel class of anti-RNAi agents that is more effective in suppressing miRNA function on HCV than other agents, such as LNAs. These novel anti-RNAi agents directed against HCV RNA ameliorated outcome in HCV replicon cells. These findings have led us to conclude that iMIRs may serve as novel, anti-RNAi therapeutic agents that are potentially safe and are excellent candidates for testing in clinical applications.

## Materials and methods

**Figure 7:**
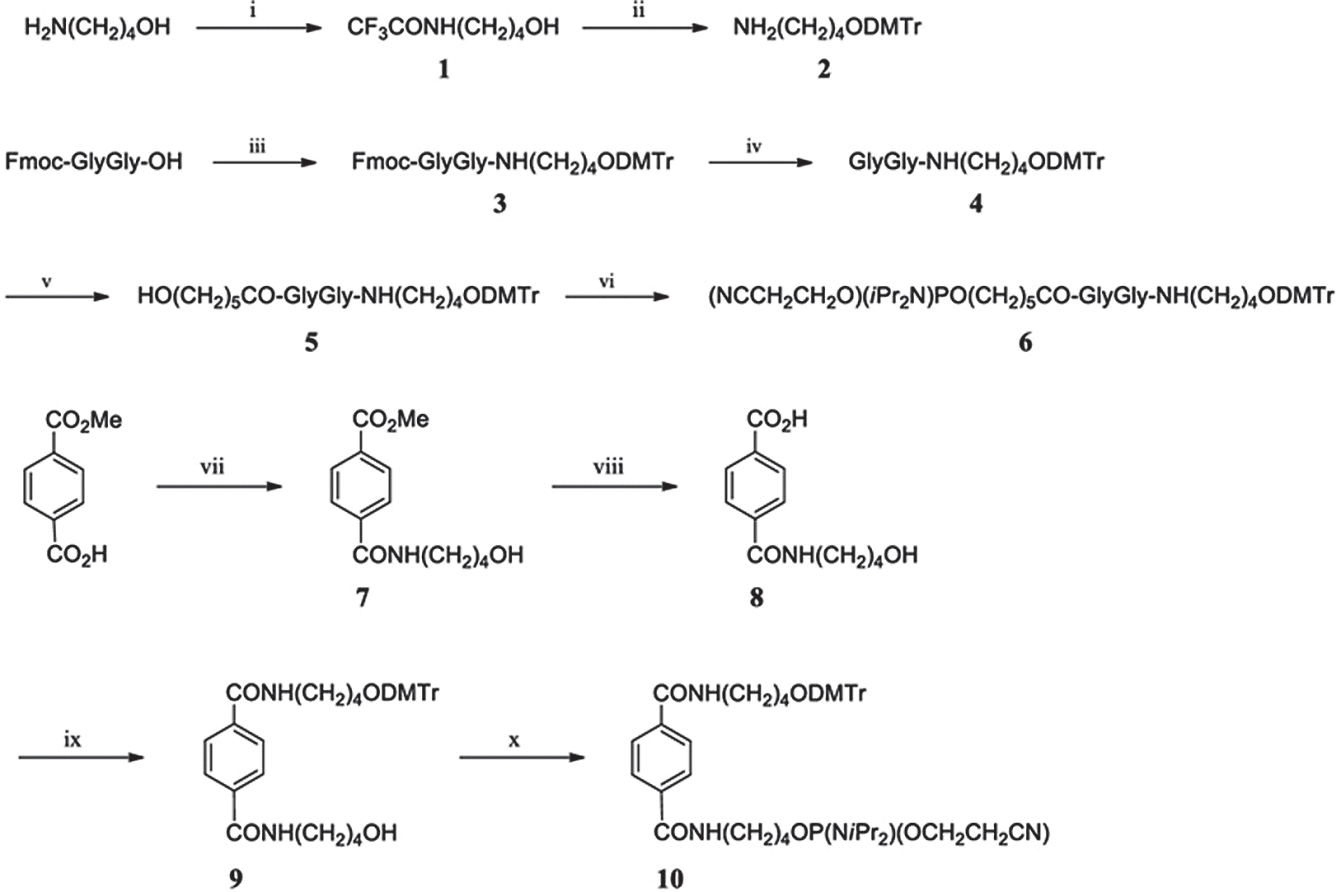
**The synthesis of glycyl-glycine diamide amidite (6) and terephthalate diamide amidite (10)**. (i); CF_3_CO_2_Et/EtOH (quant.); (ii) 1) DMTrCl/Pyridine; 2) NaOH/MeOH (quant. for 2 steps); (iii) **2**/DCC/HOBt/DMF; (iv) Piperidine/DMF; (v) 6-Hydroxyhexanoic acid/DCC/HOBt/DMF (69% for 3 steps); (vi) 2-Cyanoethoxy-*N*,*N*,*N*′,*N*′-tetraisopropylphosphorodiamidite/ diisopropylammonium tetrazolide/CH_3_CN (82%); (vii) 4-Aminobutanol/DCC/HOBt/TEA/THF (65%); (viii) NaOH/MeOH (89%); (ix) **2**/DCC/HOBt/TEA/DMF (90%); (x) 2-Cyanoethoxy-*N*,*N*,*N*′,*N*′-tetraisopropylphosphorodiamidite/ diisopropylammonium tetrazolide/CH_2_Cl_2_ (93%).

*Preparation of glycyl-glycine diamide amidites and terephthalate diamide amidites.* Glycyl-glycine diamide amidites and terephthalate diamide amidites were synthesized and incorporated into suitable positions within long RNA oligomers. Synthesis of these amidites was performed following the scheme described in **Figure 7**. 4-(4,4′-Dimethoxytrityloxy)butylamine (**2**) was prepared from 4-amino-1-butanol. Fmoc-Gly-Gly-OH and amine **2** were treated with dicyclohexylcarbodiimide (DCC) and 1-hydroxybenzotriazole monohydrate (HOBt) to create a condensated product (**3**). The Fmoc group was then removed using piperidine to obtain *N*-(4-(4,4′-dimethoxytrityloxy)butyl)-glycylglycinamide (**4**). The dehydrated compound **4** was combined with 6-hydroxyhexanoic acid, DCC, and HOBt led to *N*-(4-(4,4′-dimethoxytrityloxy)butyl)-*N*^*α*^-(6-hydroxyhexanoyl)-glycylglycinamide (**5**). Finally, compound **5** was converted into the corresponding phosphoramidite, glycyl-glycine diamide amidite (**6**). Terephthalate diamide amidite was synthesized from monomethyl terephthalate. This starting material and 4-amino-1-butanol were treated with DCC, HOBt, and triethylamine to create the condensated product (**7**), and then methoxycarbonyl group was hydrolyzed to obtain carboxylic acid **8**. Condensated carboxylic acid **8** and amine **2** were dehydrated with DCC, HOBt, and triethylamine, which yielded *N*-(4-hydroxybutyl)-4-(4-(4,4′-dimethoxytrityloxy)butylcarbamoyl)benzamide (**9**). Finally, compound **9** was converted into the corresponding phosphoramidite, terephthalate diamide amidite (**10**).

*Preparation of iMIR agents.* iMIRs were composed of phosphodiester-containing oligonucleotides. DNA and RNA oligonucleotides containing non-nucleotide residue were synthesized using commercially available controlled pore glass solid supports placed in columns that were installed in an Applied Biosystems 3900 nucleic acid synthesizer (Life Technologies, Carlsbad, CA). DNA and RNA oligomers lacking non-nucleotide residues were synthesized using standard phosphoramidites. Glycyl-glycine or terephthalate residues were incorporated into the oligomers using glycyl-glycine diamide amidite or terephthalate diamide amidite generated as described above (**Figure 7**). In the coupling reactions, trichloroacetic acid in dichloromethane was used as a detritylation agent, and amidites in acetonitrile and 5-benzylmercaptotetrazole were used as activating reagents. All commercially available reagents and solvents were used without further purification. Protecting groups were removed from oligonucleotides after the synthesis reactions were completed. The purity of the final products was confirmed by anion-exchange HPLC, using the Shimadzu LC-10A system (Shimadzu Corporation, Kyoto, Japan) equipped with a DNAPac PA-100 column (4 × 250 mm: DIONEX, Sunnyvale, CA) and by polyacrylamide gel electrophoresis. Mass spectrometry analysis was performed by liquid chromatography (ACQUITY UPLC, IonBench, Joigny, France) coupled with electrospray ionization-quadrupole-time-of-flight tandem mass spectrometry (LC-ESI-Q-TOF/MS) (SYNAPT G2 MS, Waters Corporation, MA). Following ethanol precipitation, purified oligomers were dissolved in nuclease-free distilled water (**Supplementary Materials and Methods**).

*Cell culture and transfection.* OR6 and HepG2 cells were cultured in Dulbecco's modified Eagle's medium (Wako, Osaka, Japan) containing 10% fetal bovine serum and nonessential amino acids (Life Technologies).^19^ OR6 cells carried the HCV genotype 1b replicon and a *Renilla* luciferase reporter gene, and selective pressure was maintained by including 300 μg/ml G418 (Nacalai Tesque, Kyoto, Japan) in the culture medium. OR6 cells were seeded the day before transfection in 12-well collagen-coated plates (ASAHI GLASS, Tokyo, Japan) and grown to 50% confluency. Nonspecific siRNA (Bonac, Kurume, Japan), double-stranded mature miRNA (Bonac), ASO-miR-122 (Hokkaido System Science, Sapporo, Japan), locked nucleic acid (LNA)-miR-122 (Exiqon, Vedbaek, Denmark), or iMIRs (Bonac) were transfected at a final concentration of 40 nmol/l into OR6 cells with RNAiMAX (Life Technologies).

*HCV replicon quantitative assay measuring luciferase activity.* For the dual luciferase assays, 5 × 10^4^ OR6 cells were seeded in 12-well plates in at least triplicate wells for each assay and cultured for 24 hours. The cells were cotransfected for 24, 48, and 72 hours with 1 μg/ml of a pGL3 firefly luciferase control vector (Promega, Madison, WI) and oligonucleotides or iMIRs (40 nmol/l). Cells were then harvested with passive lysis buffer (Promega) and analyzed in Dual-luciferase Reporter Assay System according to the manufacturer's suggested protocol (Promega). *Renilla* luciferase activity was normalized by comparison to firefly luciferase activity.

*HCV replicon RNA measurements by real-time qPCR.* Total RNA was extracted from harvested cells using Sepasol-RNA I Super G Solution (Nacalai Tesque). The 5′-UTR of HCV genomic RNA was converted to cDNA with a primer (5′-TGCTCATGGTGCACGGTCTA-3′) specific for the HCV5′-UTR and the TaqMan Reverse Transcription Reagents Kit (Life Technologies). Using this approach, cDNA from serially diluted HCV RNA standards (10^2^ to 10^8^ copies/well) and experimental samples was generated for qPCR. Real-time PCR reactions were performed using the Fast Start Universal Probe Master Mix (Roche, Basel, Switzerland) and a StepOnePlus Real-Time PCR System (Life Technologies). The sequences of PCR primer and probes used for qPCR studies are as follows: HCV 5′UTR Fw: 5′-CGGGAGAGCCATAGTGG-3′, HCV 5′UTR Rv: 5′-AGTACCACAAGGCCTTTCG-3′, HCV 5′UTR probe: 5′-FAM-CTGCGGAACCGGTGAGTACAC-TAMRA-3′.^45^

*Real-time qPCR tests for measuring interferon-related-gene responses.* To detect interferon-related genes such as interferon-stimulated gene 15 (ISG15) and retinoic acid-inducible gene 1 (RIG-I), cDNAs were synthesized with random hexamer primers using the Transcriptor First Strand cDNA Synthesis Kit (Roche), and real-time qPCR was performed with the Fast Start Universal SYBR Green Master (Roche). The sequences of primer used are as follows: ISG15; Fw: 5′-AGCGAACTCATCTTTGCCAGTACA-3′, Rv: 5′-CAGCTCTGACACCGACATGGA-3′, RIG-I; Fw: 5′-AAAGCATGCATGGTGTTCCAGA-3′, Rv: 5′-TCATTCGTGCATGCTCACTGATAA-3′, β-actin; Fw: 5′-CCACTGGCATCGTGATGGAC-3′, Rv: 5′-TCATTGCCAATGGTGATGACCT-3′.

*Immunoblot analysis.* OR6 cells were homogenized in 50 mmol/l Tris-HCl (pH 8.0), 150 mmol/l NaCl, 0.1% sodium dodecyl sulfate, 0.5% deoxycholic acid, and 1% NP-40. Cellular debris was removed by centrifugation, and supernatants were boiled and mixed with an equal volume of 20% glycerol containing 0.02% bromophenol blue. Proteins were resolved on 7 or 10% sodium dodecyl sulfate–polyacrylamide gel electrophoresis gels, and transferred to polyvinylidene difluoride membranes (Millipore, Billerica, MA). Membranes were incubated with primary antibodies against the HCV core antigen (Clone C7-50: Abcam, Cambridge, UK) and GAPDH (Clone sc-25778: Santa Cruz, Dallas, TX), and horseradish peroxidase-conjugated goat anti-rabbit or anti-mouse immunoglobulin G antibodies (GE Healthcare, PA) were used as secondary antibodies. Immunoreactive bands were visualized by enhanced chemiluminescence (GE Healthcare).

*Cell proliferation assays.* The Cell Proliferation Kit II (XTT: Roche) was used to evaluate potential toxicity resulting from transfecting cells with oligonucleotides or iMIRs, following the manufacturer's instructions. Briefly, OR6 cells (1 × 10^4^ cells/well) were seeded into 96-well plates, and cells were transfected with 4 pmol of each oligonucleotide or iMIR, using Lipofectamine RNAiMAX (Life Technologies). At 48 hours post-transfection, 50 µl of XTT labeling mixture was added to wells and cells were then incubated in a humidified atmosphere for 6 hours at 37 °C. Subsequently, absorbance was measured in an enzyme-linked immunosorbent assay plate reader at 450–500 nm, using a reference wavelength of 650 nm.

*Statistical analysis.* Data were analyzed statistically by the Student's *t*-test and *P* values less than 0.05 were considered statistically significant.

**SUPPLEMENTARY MATERIAL** **Figure S1.** Resistance of iMIRs to nuclease digestion. **Supplementary Materials and Methods**

## Figures and Tables

**Table 1:**
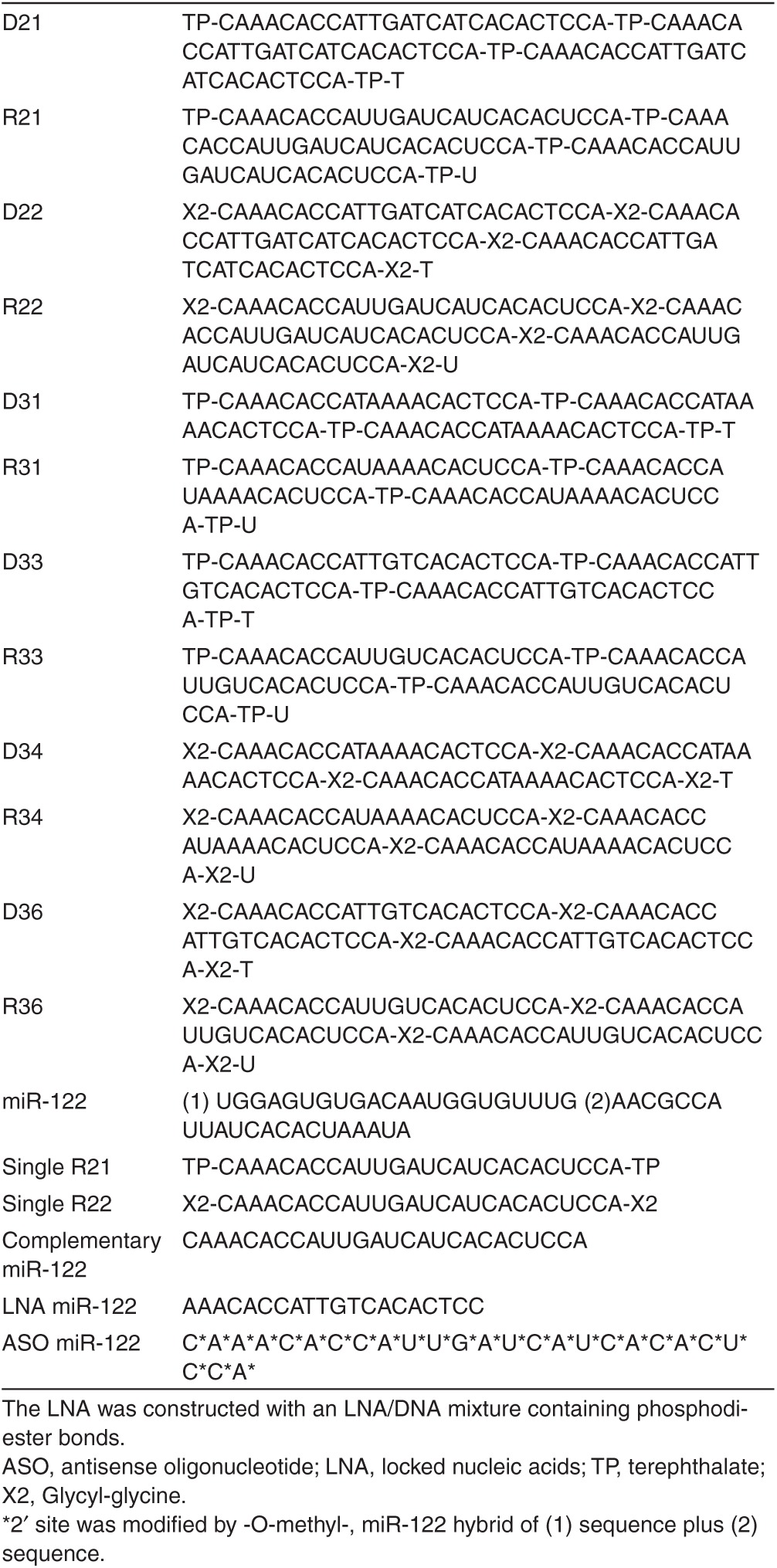
List of oligonucleotide sequences of iMIRs, ASOs, LNAs, and related miRNAs

## Supplementary Material

Supplementary Figure S1 Resistance of iMIRs to nuclease digestion.
