# Supplementary figures and images for: Control of HCV Replication With iMIRs, a Novel Anti-RNAi Agent
Source: Mol Ther Nucleic Acids. 2015 Jan 20;4(1):e219–. doi: 10.1038/mtna.2014.71 (PMC4345303; doi:10.1038/mtna.2014.71)

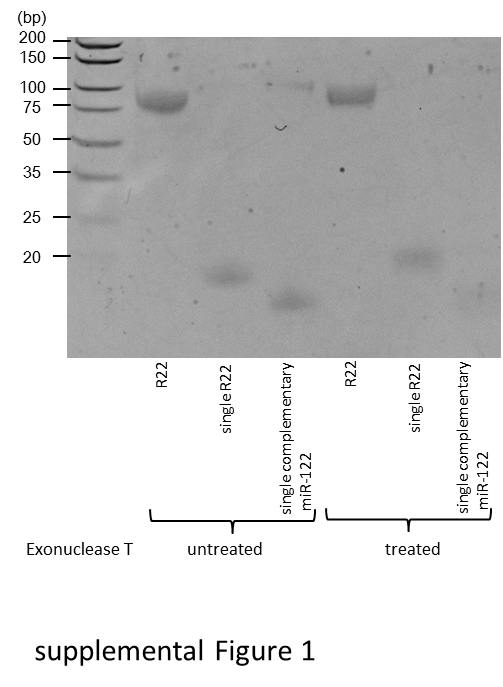

Supplement: Supplementary Figure S1 — Resistance of iMIRs to nuclease digestion. [file mtna201471x1.tiff]
