# Supplementary material for: Control of HCV Replication With iMIRs, a Novel Anti-RNAi Agent
Source: Mol Ther Nucleic Acids. 2015 Jan 20;4(1):e219–. doi: 10.1038/mtna.2014.71 (PMC4345303; doi:10.1038/mtna.2014.71)
Supplement: Supplementary Materials and Methods [file mtna201471x2.doc]

Supplementary Materials and Methods

The indicated oligonucleotides (5μg each) were treated with 2.5 U Exonuclease T (New England BioLabs, Ipswich MA) according to the manufacturer’s suggested protocol in a 30-μL reaction mixture for 30 min at 25ºC. Exonuclease T treated iMIR was separated on a 20% polyacrylamide gel containing 7M urea. After electrophoresis at 150V for 120 min in TBE buffer, the gel was stained with Midori Green (Nippon Genetics), and oligonucleotides were detected under UV light.
